# Accelerating carrier separation to boost the photocatalytic CO_2_ reduction performance of ternary heterojunction Ag–Ti_3_C_2_T_*x*_/ZnO catalysts†

**DOI:** 10.1039/d4ra01985g

**Published:** 2024-04-25

**Authors:** Qilin Han, Zhiyao Wu, Yu Zhou, Yongxin Lei, Bingying Nie, Leilei Yang, Wenbin Zhong, Nannan Wang, Yanqiu Zhu

**Affiliations:** a State Key Laboratory of Featured Metal Materials and Life-cycle Safety for Composite Structures, MOE Key Laboratory of New Processing Technology for Nonferrous Metals and Materials, School of Resources, Environment and Materials, Guangxi University Nanning 530004 China wangnannan@gxu.edu.cn; b College of Mathematics and Physics, Guangxi Minzu University Nanning 530006 China yangllei@gxmzu.edu.cn

## Abstract

Developing low-cost and efficient photocatalyst/co-catalyst systems that promote CO_2_ reduction remains a challenge. In this work, Ag–Ti_3_C_2_T_*x*_ composites were made using a self-reduction technique, and unique Ag–Ti_3_C_2_T_*x*_/ZnO ternary heterojunction structure photocatalysts were created using an electrostatic self-assembly process. The photocatalyst's close-contact heterogeneous interface increases photogenerated carrier migration efficiency. The combination of Ti_3_C_2_T_*x*_ and Ag improves the adsorption active sites and reaction centers for ZnO, making it a key site for CO_2_ adsorption and activation. The best photocatalysts had CO and CH_4_ reduction efficiencies of 11.985 and 0.768 μmol g^−1^ h^−1^, respectively. The CO_2_ conversion was 3.35 times better than that of pure ZnO, which demonstrated remarkable stability even after four cycle trials with no sacrificial agent. Furthermore, *in situ* diffuse reflectance infrared Fourier transform spectroscopy (*in situ* DRIFTS) and valence band spectroscopy were utilized to propose the photocatalytic reaction mechanism and electron transfer channels of the Ag–Ti_3_C_2_T_*x*_/ZnO system, confirming that CHO* and CO* are the important intermediates in the generation of CH_4_ and CO. This study introduces a novel method for the development of new and efficient photocatalysts and reveals that Ti_3_C_2_T_*x*_ MXene is a viable co-catalyst for applications.

## Abstract 2

Developing low-cost and efficient photocatalyst/co-catalyst systems that promote CO_2_ reduction remains a challenge.

## Graphical abstract

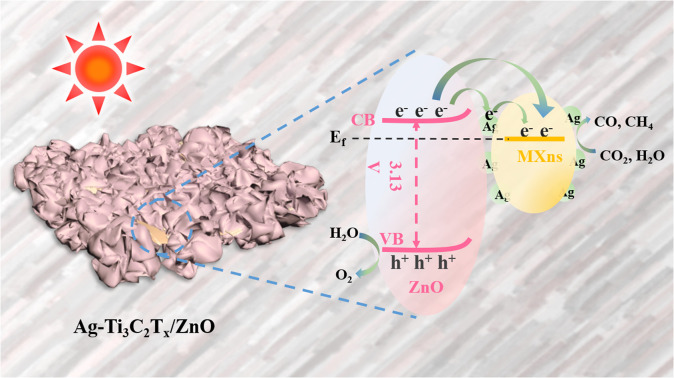

## 1. Introduction

With the continuous advancement of urbanization and industrialization, environmental pollution and energy shortage problems are deteriorating.^1–4^ Photocatalytic CO_2_ reduction technology is an environmentally friendly clean fuel production technology, which can not only effectively reduce CO_2_ emissions, but also alleviate the energy shortage problem.^5–7^ However, common single semiconductor photocatalysts have defects such as wide bandgap, fast photogenerated carrier complexation rate, and low visible light utilization efficiency. Therefore, researchers have compensated for the shortcomings of single semiconductors by elemental doping, co-catalyst loading, and constructing heterojunctions to improve the activity of photocatalysts.^8–11^ Precious metal nanoparticles (Au, Ag, Pt) are widely used in enhancing the photocatalytic activity of semiconductors due to their localized surface plasmon resonance properties.^12,13^ Ag nanoparticles can enhance the photocatalytic activity by exciting the localized surface plasmon into the conduction band of the semiconductor material. However, the contact area between the metal nanoparticles and the semiconductor material is small, which is not conducive to electron migration and high carrier recombination efficiency. The development of low-cost and efficient photocatalyst/co-catalyst systems to enhance the photocatalytic reduction of CO_2_ remains a challenge.

Two-dimensional transition metal carbide–nitride and carbon–nitride MXenes materials have attracted a remarkable amount of attention in the materials field since their discovery in 2011.^14,15^ They are produced by selective etching of metal-bonded layered solids. Among the MXenes materials, the easiest to prepare and most widely used is Ti_3_C_2_T_*x*_, which has metallic properties and a larger work function to improve the migration and separation efficiency of photogenerated carriers.^16–18^ Meanwhile, extensive surface-active sites and vacancies (Ti) are available on Ti_3_C_2_T_*x*_.^19^ Research has shown that suitable metal atoms exposed to the photocatalyst surface can act as adsorption sites and reaction sites for CO_2_, which can accept photogenerated electrons from the photocatalyst and transfer them to the reactants (CO_2_, H_2_O, and intermediates) to carry out redox reactions with enhanced separation of photogenerated electrons from holes.^20–23^ Therefore, Ti_3_C_2_T_*x*_ shows great potential for application as a photoredox co-catalyst in clean energy conversion. Hong *et al.*^24^ prepared BiOIO_3_/g-C_3_N_4_ Z-type heterojunctions modified by Ti_3_C_2_ nanosheets, which significantly enhanced the efficiency of photocatalytic generation of CO and CH_4_ by the formed Z-type heterojunctions as well as the modification of two-dimensional Ti_3_C_2_ co-catalysts, and the CO yield was 6.6 times higher than that of bare g-C_3_N_4_. The nanoscale materials with a larger specific surface area are more favorable for the preparation of MXene/semiconductor composites with strong interfacial contacts.

ZnO is a direct bandgap semiconductor with a bandgap width of 3.37 eV and has superior photocatalytic activity in the ultraviolet region, which means that most of the sunlight cannot be fully utilized, seriously affecting the photocatalytic efficiency of ZnO photocatalysts.^25–27^ In addition, the rapid complexation of carriers is also an important reason hindering the improvement of the photocatalytic activity of ZnO. Therefore, for ZnO-based photocatalysts, expanding their visible light absorption range and promoting the separation of photogenerated electron–hole pairs can be beneficial to enhance their photocatalytic performance.^26^ The common pure ZnO in particle or rod form exhibited large size, fewer surface active sites, and poor photogenerated charge separation efficiency, which as a result led to poor photocatalytic performance.^28^ Hence, in attempt to solve the above problems, the investigation was carried out to enhance the photocatalytic reduction performance by changing the morphology size of ZnO or adding various co-catalysts. Li *et al.*^29^ synthesized ZnS/ZnO hollow spherical heterojunction photocatalysts by fine control of the nano/microstructure. Local lattice distortion was introduced on the surface of hollow ZnS/ZnO microspheres to activate lattice oxygen and obtain additional active reaction sites. Moreover, the construction of ZnS/ZnO heterojunctions accelerated the separation of photo-promoted carriers and significantly enhanced the photocatalytic CO_2_ reduction performance, and the photocatalysts showed a CO yield of up to 35.85 μmol g^−1^ h^−1^ under the irradiation of a 300 W xenon lamp.

In this work, Ag–Ti_3_C_2_T_*x*_ composites and Ag–Ti_3_C_2_T_*x*_/ZnO (ATZ) ternary heterojunction structure were prepared by utilizing Ti_3_C_2_T_*x*_ self-reducing properties and electrostatic self-assembly technique. The catalysts possessed superior photocatalytic activity. The construction of ternary heterojunction effectively enhanced the separation and migration of photogenerated carriers. The larger specific surface area of the two-dimensional material and the exposed metal atoms on the surface provided more active sites for CO_2_ adsorption. Meanwhile, in the ATZ system, both Ag and Ti_3_C_2_T_*x*_ can be employed as electron traps to capture electrons in ZnO, accelerating the migration of photogenerated electron–hole pairs and enlarging the photocatalytic CO_2_ reduction efficiency. We believe that this study will provide a new pathway for the preparation of high-performance semiconductor photocatalysts and new MXene-based composite photocatalysts.

## 2. Experimental

### 2.1 Materials

Ti_3_AlC_2_ powder (98 wt%) was obtained from China Forsman Technology Co., Ltd. LiF powder (98 wt%) was purchased from Sinopharm Group Chemical Reagent Co., Ltd. Zinc acetate hexahydrate and hydrochloric acid were analytically pure reagents purchased from Guangdong Guanghua Technology Co., Ltd. Sodium hydroxide powder was purchased from Shanghai Aladdin Reagent Co., Ltd. Cetyltrimethylammonium bromide (CTAB) was an analytically pure reagent purchased from Tianjin Damao Chemical Reagent Factory. The water used in the experiments was ultrapure.

### 2.2 Preparation of Ti_3_C_2_T_*x*_ nanosheets

1 g of LiF was dispersed in 20 mL of HCl solution (9 M) in a polytetrafluoroethylene liner with uniform stirring for 10 min. Subsequently, 1 g of Ti_3_AlC_2_ (MAX) powder was slowly added to the above solution in several portions, and the resulting mixed solution was stirred in an oil bath (36 °C) for 48 h. The solution was then washed by repeated centrifugation (8000 rpm, 5 min) until the pH was ≥6. After, the black precipitate was taken and freeze-dried for 48 h to obtain organoid Ti_3_C_2_T_*x*_ (MX).^30^ The appropriate amount of deionized water was added to the MX powder and sonicated for 2–4 h under the environment of the ice water bath, followed by centrifugation at 8000 rpm for 5 min, and the dark-green supernatant was taken and dispensed into a Petri dish and freeze-dried for 72 h, to obtain the less-layered Ti_3_C_2_T_*x*_ nanosheets (MXns).

### 2.3 Preparation of Ag–Ti_3_C_2_T_*x*_

Ag–Ti_3_C_2_T_*x*_ was prepared by the self-reduction strategy, which is abbreviated as *x*AT according to the difference in the mass percentage of Ag added, as follows. 20 mg MXns was taken into 20 mL of water and sonicated for 30 min, 2 mL of 0.05 mg mL^−1^ of AgNO_3_ solution was added to the solution, stirred for 6 h, and freeze-dried for 24 h to obtain 0.5AT. Different contents of Ag–Ti_3_C_2_T_*x*_ were prepared by adjusting the volume of AgNO_3_ solution (5 and 10 mL), which were distinguished as 1AT and 2AT.

### 2.4 Preparation of ZnO nanoflower

5.96 g of Zn (NO_3_)_2_·6H_2_O was added into 45 mL of ultrapure and ultrasonicated for 10 min until it was completely dissolved to produce solution A. Then 2.4 g of NaOH was incorporated into 15 mL of water to produce liquor B. Subsequently, liquor B was injected into solution A, and the solution was stirred vigorously at 90 °C for 2 h to obtain the ZnO nanoflowers.

### 2.5 Preparation of Ag–Ti_3_C_2_T_*x*_ MXene/ZnO

Ag–Ti_3_C_2_T_*x*_ MXene/ZnO composites were prepared by electrostatic self-assembly method. First, 200 mg of surfactant CTAB was added to 200 mL of deionized water and stirred thoroughly for 10 min, and then 30 mg of ZnO was added and stirred for 3 h to obtain solution 1. Under the action of ultrasonication, 5 mg of AT was sufficiently dispersed into 20 mL of water to form suspension 1. Suspension 1 was added into solution 1, stirred for 3 h, rinsed with water, and freeze-dried for 24 h. The ATZ composite sample was finally obtained. To better distinguish the samples, they were named *x*ATZ (*x* denotes the mass percentage of Ag, *x* = 0.5, 1 and 2). The ZnO/Ti_3_C_2_T_*x*_ (*ZT*) composite samples were prepared in the same way to facilitate subsequent comparative testing.

### 2.6 Characterization

The constituent elements of the material were analyzed using an X-ray diffraction instrument (XRD, Rigaku D/MAX 2500 V, Japan) equipped with Cu-Kα radiation on a Cu target at a wavelength of *λ* = 1.5406 Å. The morphology and microstructure of the samples were analyzed using a field emission scanning electron microscope (SEM, Sigma 300, CARL ZEISS, Germany) and a transmission electron microscope (TEM, TecnaiF20, FEI, USA). The catalysts were further characterized for structural changes using Fourier Transform Infrared Spectroscopy (FTIR, Nicolet iS50, Thermo Fisher Scientific, USA) and Laser Raman Spectroscopy (Raman, inVia Rdflex, Renishaw, UK). Laser Raman spectra were recorded by a Leica DM2700 microscope at a wavelength of 512 nm and a maximum output power of 5 mW. The surface structure and surface charge transfer of the materials were analyzed using an X-ray photoelectron spectrometer (XPS, ESCALAB 250Xi, Thermo Fisher, USA). The N_2_ adsorption–desorption isothermal curves were tested using an automated specific surface area analyzer (BET, ASAP 2020, Micromeritics, USA) and all the samples were Degassing was carried out at 150 °C for 12 h. UV-visible diffuse reflectance spectra in the range of 200–800 nm were recorded using a UV/Vis spectrophotometer (DRS, UV-3600Plus, SHIMADZU, Japan). Photoluminescence spectra (PL, FL3C-111 TCSPC, HORIBA, Japan) was obtained to study carrier separation of samples.

### 2.7 Photocatalytic CO_2_ reduction reaction

The photocatalytic reduction of CO_2_ test was carried out in a photoreactor with a total capacity of 150 mL. Firstly, 10 mg of photocatalyst was homogeneously dispersed in 800 μL of ethanol solution, and then it was coated on a 2.5 cm × 2.5 cm glass slice, which was dried in a vacuum drying oven for 4 h. 20 mL of 0.1 M NaHCO_3_ solution was placed in the reaction cell, and the glass slice was placed on the high platform of the reactor to avoid contacting the solution. The top of the reaction cell was sealed with rubber clamps, and high-purity CO_2_ was introduced into the reaction cell and bubbled for 30 min. Finally, the photocatalytic reduction test was completed under the irradiation of a 300 W xenon lamp (PLS-SXE300D, Perfectlight, China) with the light intensity of 100 mW cm^−2^. Recirculating water was passed through the entire reaction process to ensure a constant test temperature. After the photocatalytic reaction, a gas bag driven by a peristaltic pump was used to collect the gases in the photoreactor and injected into a gas chromatograph (GC-2014C, Shimadzu, Japan) for gas analysis, and the liquid products were filtered and then detected and analyzed by an autosampler (AOC-20i, Shimadzu, Japan).

### 2.8 Photoelectrochemical measurements

The photocurrent response, electrochemical impedance spectra, and Mott–Schottky curves were tested using an electrochemical workstation with a three-electrode system (CHI660E, Chenhua, Shanghai, China). Pt electrode was used as the counter electrode and an Ag/AgCl electrode was used as the reference electrode. The working electrode was prepared as follows: 10 mg of sample was dispersed in 0.2 mL of anhydrous ethanol and 20 μL of Nafion solution to form a uniform electrode slurry. 50 μL of the dispersion was taken and coated on a 1 cm × 1 cm fluorine-doped tin oxide (FTO) conductive glass. A 300 W Xe lamp was used as a light source and 0.5 M Na2SO_4_ was used as the electrolyte solution.

## 3. Results and discussion

### 3.1 Synthesis, structure, and morphology

**Fig. 1:**
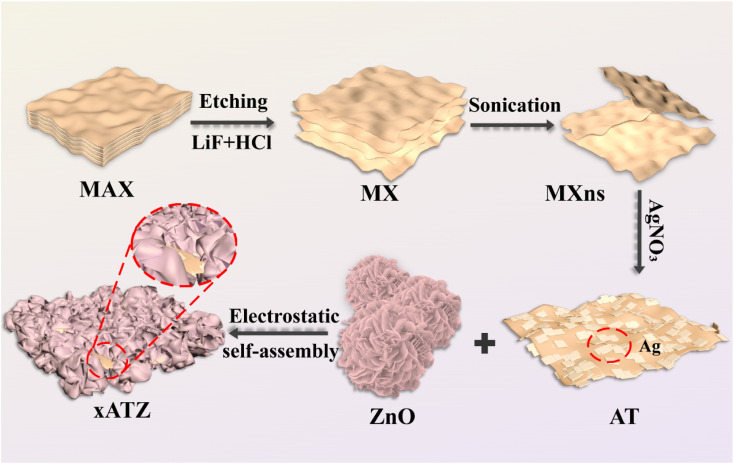
Schematic illustration of the synthesis of MZx composite.

The preparation process of *x*ATZ ternary heterojunction composite photocatalyst is shown in Fig. 1. Initially, HCl and LiF were utilized for *in situ* etching, and the few-layer MXns were obtained after ultrasonication in an ice-water bath. The reaction equations are as follows:^24^

1 HCl + LiF = LiCl + HF

2 3HF + Ti_3_AlC_2_ = Ti_3_C_2_ + AlF_3_ + 3/2H_2_

MXns was homogeneously dispersed in water, and then AgNO_3_ solution was added to produce ATZ composite photocatalysts loaded with singlet Ag on the surface of MXns, leveraging the strong reducing property of the MXns surface. Then, the cationic surfactant CTAB was used to make the ZnO precursor surface loaded with a positive charge. Owing to the negative electric properties of MXns and the opposite charge of ZnO, the combination of positive and negative charges synthesized the *x*ATZ composites by electrostatic self-assembly.^20,31,32^

**Fig. 2:**
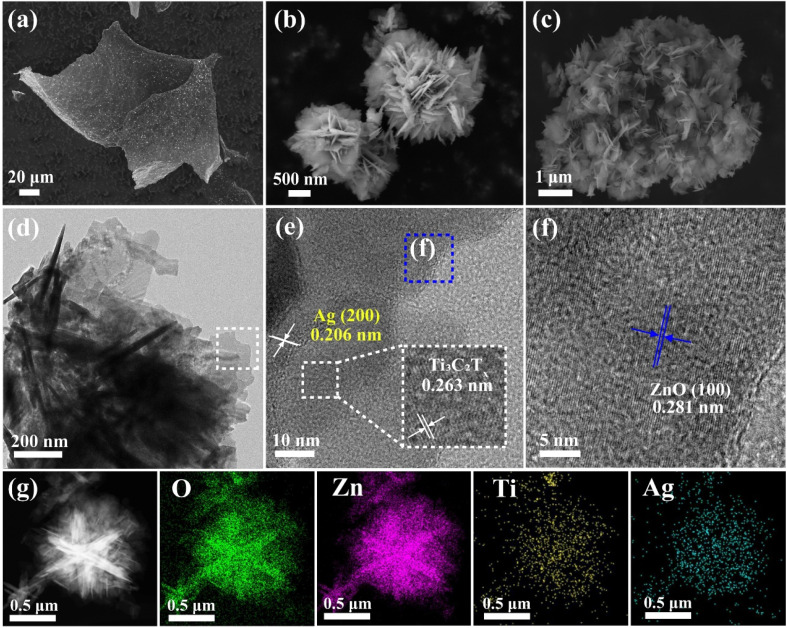
The SEM spectras of (a) MXns, (b) ZnO and (c) 1ATZ. (d) TEM image of 1ATZ. (e and f) HR-TEM images of 1ATZ. (g) Elemental mapping of O, Zn, Ti, and Ag.

The materials were analyzed morphologically using scanning electron microscopy. Fig. 2a shows the few-layer titanium carbide nanosheets (MXns) obtained from Ti_3_AlC_2_ after HF etching and Li^+^ intercalation, and continuous sonication in an ice-water bath for 2 h. The Ti_3_AlC_2_ is a lumpy morphology with a delamination tendency, whereas the un-sonicated MX is an accordion-like shape with a large spacing of layers (Fig. S1†), which is a change in the morphological characteristics that are corroborated with the characterization results of XRD.^14^ The nanoflower-like ZnO was obtained after morphology adjustment, as shown in Fig. 2b. As shown in Fig. S2a,† different amounts of Ag clusters were burdened on the surface of MXns through the self-reduction of AgNO_3_, providing more active sites for CO_2_ adsorption for the photocatalytic reduction process. The dispersion of Ag clusters on the MXns surface was further demonstrated by SEM-EDS. The AT and ZnO were coupled by electrostatic self-assembly strategy to form *x*ATZ composite samples, and it can be observed from Fig. 2c that the ZnO nanoflowers were uniformly loaded onto the AT to form a lamellar stacked nanosheet state, which in turn increased its specific surface area.

The morphology and structure of the materials were further analyzed by transmission electron microscopy. From Fig. 2e, it can be observed that the nanoflowers and ultrathin nanosheets are coupled while forming well-defined interfaces at the junctions, proving the successful preparation of the composite samples. The presence of ultrathin nanosheets indicates that Ti_3_AlC_2_ is sufficiently exfoliated into few-layers of MXns. Fig. 2(e and f) show the HRTEM images of the 1ATZ, with lattice fringes with lattice spacings of 0.263 nm and 0.281 nm attributed to the MXns (0 1̄ 1 0) crystal plane of MXns and (1 0 0) crystal plane of fibrillated zincite ZnO, respectively, consistent with XRD characterization.^22,33^ In addition, the lattice spacing of 0.206 nm is consistent with the (2 0 0) crystalline plane of Ag, and the appearance of this lattice stripe proves the successful self-reduction of AgNO_3_ on the surface of MXns. The EDS elemental mapping of Ti, Zn, Ag, and O is shown in Fig. 2g, which further confirms the close contact and positional relationship of ZnO and AT in 1ATZ composite samples, and ZnO is tightly loaded on the AT substrate.

**Fig. 3:**
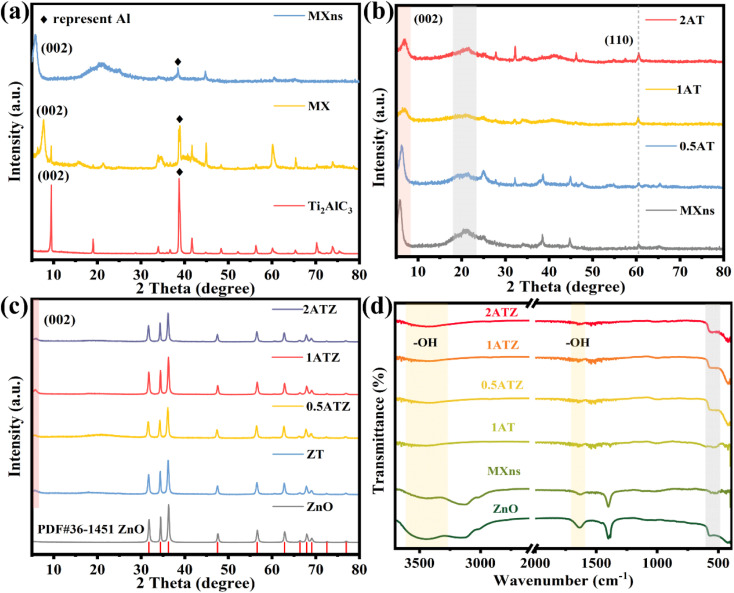
XRD patterns of (a) MXns, MX and Ti_3_AlC_2_, (b) MXns and *x*AT, (c) ZnO and *x*ATZ. FT-IR spectra of (d) ZnO, MXns, and *x*ATZ.

The crystal structure and physical phase characteristics of the material were analyzed by XRD. As shown in Fig. 3a, the changes in the physical phase composition of the titanium carbide material before and after etching were displayed. The diffraction peaks located at 39° attributed to the Al (104) facet gradually weakened after HF etching of Ti_3_AlC_2_ (MAX), and the diffraction peaks almost completely disappeared when few/monolayer MXns were formed, which proved the successful etching of the Al layer in Ti_3_AlC_2_.^21^ The diffraction peak belonging to the (002) crystal plane is gradually shifted to the left from 9° to 5.9° after etching, and at the same time, its diffraction peak is broadened, which proves that the layer spacing of the material is gradually increased and the MXns is successfully prepared.^34^ As shown in Fig. 3b, the composite *x*AT after self-reducing Ag on the surface of MXns maintains the original skeleton of the two-dimensional material, indicating that the introduction of metallic Ag did not destroy the crystal structure of the raw material. However, no characteristic diffraction peaks about Ag appeared in the pattern, which may be due to the low content of added Ag and weak crystallinity. Fig. 3c shows the XRD diffraction pattern of the *x*ATZ composite sample. By comparing the standard PDF cards, most of the diffraction peaks of *x*ATZ coincide with the hexagonal structure of ZnO (Hexagonal, *P*6_3_*mc*), and there are three strong diffraction peaks at 31.77°, 34.42°, and 36.26°, which correspond to the (100), (002), and (101) of the ZnO crystal faces (PDF#36-1451).^35^ A characteristic peak belonging to the MXns (002) crystal plane appears at 2*θ* = 5.9°, proving that the AT was successfully loaded onto ZnO.

FTIR spectroscopy can be used to analyze the spatial structure of the materials as well as the composition of the functional groups. As can be seen from Fig. 3d, all the samples have a broad absorption band at 3600–3400 cm^−1^, which can be attributed to the stretching vibration of the –OH group.^36,37^ Meanwhile, a weak characteristic peak appeared at 1640 cm^−1^, which was attributed to the deformation vibration absorption peak of the hydroxyl group on the surface of ZnO, and the peak could be generated by the adsorbed hydroxyl group formed by the final dissociation of adsorbed water on the surface of ZnO.^26^ The absorption bands observed in the range of 850–500 cm^−1^ for all the composite photocatalysts can be attributed to the Zn–O vibration.^26^ Also, a stretching vibrational absorption band at 480 cm^−1^ attributed to the Ti–C part can be observed, indicating that the MXns are retained in the ZnO structure, in other words proving the successful synthesis of the *x*ATZ material.^38^

**Fig. 4:**
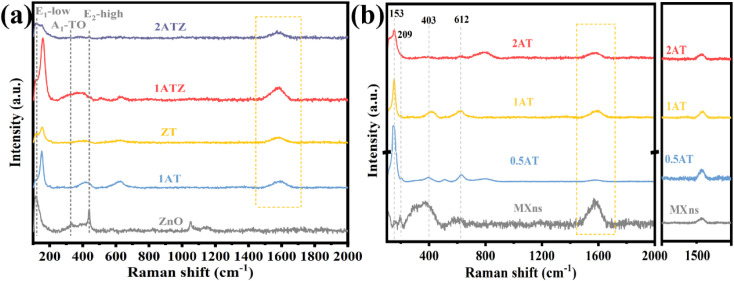
Raman spectra of (a) ZnO, 1ATZ, *ZT*, and *x*ATZ, (b) MXns and *x*ATZ composites samples.

Raman spectroscopy was further used to investigate the crystal structure of the material. It can be observed in Fig. 4a that pure ZnO has distinct characteristic peaks at 100 and 438.2 cm^−1^ corresponding to the high and low-frequency branches of the E_2_ mode (E_2_-high and E_2_-low).^39^ These two E_2_ modes attributed to oxygen sublattice and heavy-atom vibrations together constitute the representative characteristic peaks of the ZnO structure of the fibrillated zincite hexagonal crystals. The peaks located at 332.1 and 381.9 cm^−1^, on the other hand, are caused by the multiphonon scattering processes E_2_H–E_2_L and A_1_(TO) of ZnO crystals.^40^ However, spectral peaks that do not belong to the ZnO structure appear in the composite sample. Combined with the Raman spectra of MXns in Fig. 4b, results show that the characteristic peaks located at 153 cm^−1^ and 209 cm^−1^ are attributed to the in-plane vibrations of Ti and C atoms and the out-of-plane vibrations of Ti atoms.^41^ The characteristic peaks at 403 cm^−1^ and 612 cm^−1^ are attributed to the in-plane vibrations of the surface groups of the Ti atoms and the out-of-plane vibrations of C atoms.^41^ The presence of special Raman signals D and G peaks at 1350 cm^−1^ and 1580 cm^−1^ attributed to amorphous carbon, which are triggered by defects and lattice vibrations present in amorphous carbon, respectively.^22^*x*ATZ contains Raman signals of ZnO and MXns, which proves the successful preparation of the composite photocatalyst.

**Fig. 5:**
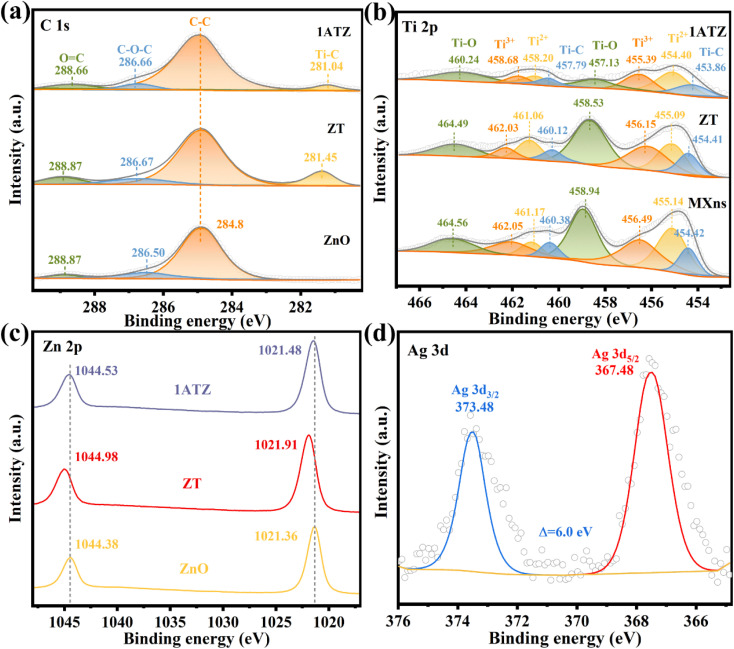
XPS spectras of ZnO, AT, *ZT*, and 1ATZ for (a) C 1s (b) Ti 2p (c) Zn 2p (d) Ag 3d.

The elemental composition of the material as well as the surface electron migration was analyzed by X-ray photoelectron spectroscopy (XPS). As shown in Fig. S3,† four elements, C, O, Ti, and Zn, are present in the 1ATZ composite, where the presence of Ti is attributed to the presence of the MXns substrate, and no obvious Ag characteristic peaks are observed in the full spectrograms due to the relatively low content of Ag. However, significant Ag signals are found in the analysis of 1AT, thus demonstrating the inclusion of five elements, C, O, Ti, Zn, and Ag, in 1ATZ as well as the successful synthesis of the composite photocatalyst. Charge correction is performed using C 1 s (284.8 eV) as the reference. Fig. 5a shows the high-resolution spectrum of C 1s. In all materials, the characteristic peaks with binding energies located at the position of 284.8 eV are attributed to the C–C of surface amorphous carbon, and the peaks at 288.87 and 286.50 eV correspond to C=O and C–O–C, respectively.^42^*ZT* and 1ATZ convolve a new peak close to the position of 281 eV (281.45 and 281.04 eV), which is attributed to the interaction between Ti and C interaction (Ti–C).^43^ In the high-resolution XPS spectra of 1ATZ on Ti, Fig. 5b, the characteristic peaks with binding energies located at 454.42, 455.14, 456.49, 458.94, 460.38, 461.17, 462.05, and 464.56 eV are fitted as C–Ti, Ti^2+^, Ti^3+^, Ti–O, C–Ti, Ti^2+^, Ti^3+^, and Ti–O.^41^ The binding energy of Ti 2p in *ZT* is shifted towards lower energy compared to MXns, indicating that the concentration of electrons around MXns is elevated and electrons are transferred from ZnO to MXns. The signals of Ti 2p in 1ATZ are significantly reduced due to the low content of MXns in 1ATZ. The change in the position of the Ti 2p binding energy between 1ATZ and MXns indicated the presence of electron transfer to MXns, further confirming the direction of the electron transfer. The characteristic peaks about Ti in AT are shifted, further demonstrating the electron transfer and strong interaction between Ag and MXns (Fig. S4†).

In the high-resolution spectra of Zn 2p (Fig. 5c), the double peaks on pure ZnO are attributed to Zn 2p^3/2^ (1021.36 eV) and Zn 2p^1/2^ (1044.38 eV) with a spin–orbit splitting fraction of 23.0 eV, suggesting the presence of oxidation state of Zn^2+^ in the near-surface region.^44^ In the 1ATZ composite sample, both peaks are shifted towards higher binding energy (1021.36 eV towards 1021.48 eV and 1044.38 eV towards 1044.53 eV), indicating a decrease in the electron density around the Zn atom. The shift in the binding energy in the composite sample suggests that the electrons are transferred from ZnO to AT due to electronic coupling at the interface. Fig. 5d shows the high-resolution XPS spectra of Ag 3d in 1AT, which can be observed that the binding energy spacing of Ag 3d is 6 eV, and the characteristic peaks with binding energies located at 367.48 and 373.48 eV are attributed to Ag 3d_5/2_ and Ag 3d_3/2_, which indicates that the AT material shouts to contain metallic Ag and Ag^+^.^45^ The presence of Ag^+^ may be attributed to the fact that some of the AgNO_3_ has not been reduced. Unfortunately, due to the low content of Ag in the 1ATZ composite, there is only a weak Ag signal in its spectrum (Fig. S5†).

**Fig. 6:**
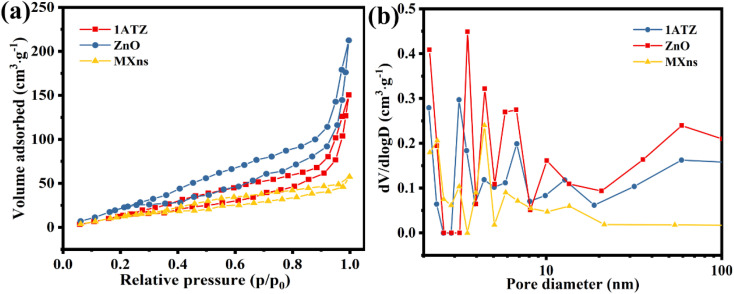
(a) N_2_ adsorption–desorption isotherms and (b) pore size distribution of 1ATZ, ZnO, and MXns.

The nitrogen adsorption–desorption curve can be used to characterize the specific surface area size and structural features of the material. From Fig. 6a, it is observed that the isotherm rises sharply in the high-pressure region and stagnates during the desorption process, forming a hysteresis line. As the relative pressure trends to 1, the curve closes and a reversible type IV isotherm for mesoporous solid generation appears,^38,46^ demonstrating that 1ATZ, ZnO, and MXns have crack-like pores, which is consistent with the lamellar morphology of the materials. The hysteresis lines are both H3 type with no obvious saturated adsorption platform and irregular pore structure.^47^ Possibly related to the stacking of flakes. The specific surface area and pore size of the samples were calculated using the BET and BJH models, and the results are shown in Fig. 6b and Table S1,† which show that 1ATZ has a larger specific surface area compared with the nano-flake MXns and nano-flower ZnO. Generally, a larger specific surface area provides more surface active sites and also facilitates the loading of other materials. Meanwhile, the average pore size of 1ATZ is smaller than ZnO, and the smaller pore size has a stronger adsorption capacity.

### 3.2 Optical properties and energy band structure

**Fig. 7:**
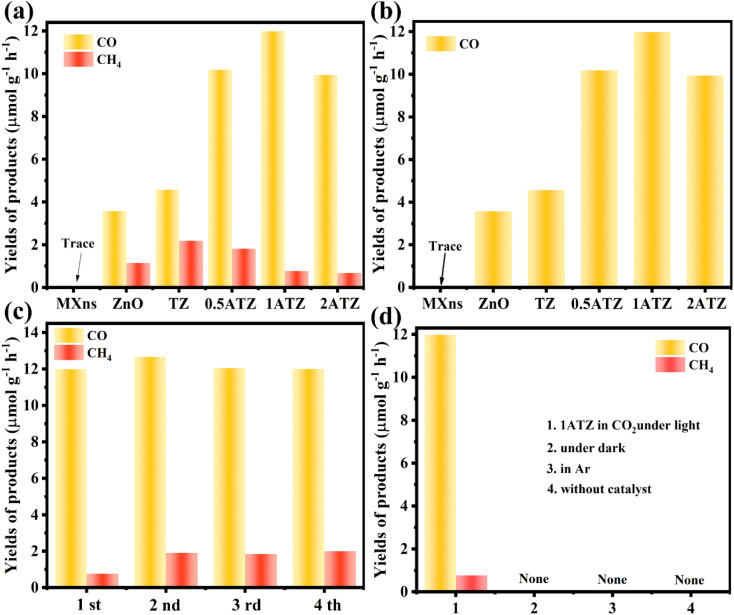
(a) Photocatalytic CO and CH4 yield plots, (b) CO yield plots of MXns, ZnO, TZ, and *x*ATZ composites. (c) Cycling activity of 1ATZ for the photocatalytic CO_2_ reduction. (d) Control experiment.

The catalytic performance of the photocatalysts was evaluated by the yield of CO and CH_4_ in the photocatalytic CO_2_ reduction system. As shown in Fig. 7a, MXns are not active in CO_2_ photoreduction, the photocatalytic activity is activated by loading ZnO, and the photocatalytic yield is further improved by the reduction of Ag on the MXns surface. The pure ZnO photocatalytic reduction products are mainly CO and the yields of CH_4_ were relatively low, which were 3.577 and 1.145 μmol g^−1^ h^−1^, respectively. The photocatalytic CO_2_ reduction is significantly enhanced when MXns was used as a co-catalyst for ZnO, and it is also observed that the relative content of Ag in MXns has a significant effect on the photocatalytic performance as well as the selectivity of the photocatalytic reduction products. From Fig. S6,† it is observed that the photocatalytic CO_2_ yield of *x*AT is relatively low, indicating that the photocatalytic activity of *x*AT composites is low. The photocatalytic performance of the catalysts is significantly enhanced with the increase of the relative content of Ag. Due to the reduction of Ag on the surface of MXns, the AT has a stronger conductivity, which accelerates the migration of carriers and facilitates the separation of photogenerated carriers in the heterojunction. However, excessive reduction of monolithic silver overconsumed the metal active sites on the surface of MXns, leading to a decrease in the photocatalytic activity, which is consistent with the SEM image of 2ATZ (Fig. S7†). The optimal photocatalyst is 1ATZ with CO and CH_4_ yields of 11.985 and 0.768 μmol g^−1^ h^−1^, respectively (Fig. 7b). The catalytic activity of the composite is higher than that of most similar composite photocatalysts (Table S2†). Tests are carried out in the dark as well as in the presence of Ar, and no products are observed. These results indicate that the reaction system is initiated by photoexcitation and the CO_2_ RR product originated from CO_2_ conversion (Fig. 7d). To judge the stability of the photocatalyst, four cycling experiments were conducted on 1ATZ, which has the best reduction performance, and the yield of CO was found to be relatively stable (Fig. 7c). The samples after the cycling experiments are collected to analyze the differences before and after the reaction. The full XPS spectrum (Fig. S8a†) shows that the chemical composition of the samples does not change before and after catalysis. In the high-resolution spectrum of Ti 2p, the characteristic peaks are shifted towards low binding energy (Fig. S8c†). In the Zn 2p XPS spectrum, the characteristic peaks are shifted toward high binding energy, indicating that the transfer of electrons occurred during the reaction process and the electrons are migrated from ZnO to MXns (Fig. S8b†). Taken together, the above test results indicate that the 1ATZ photocatalyst has strong stability in CO2 RR.

**Fig. 8:**
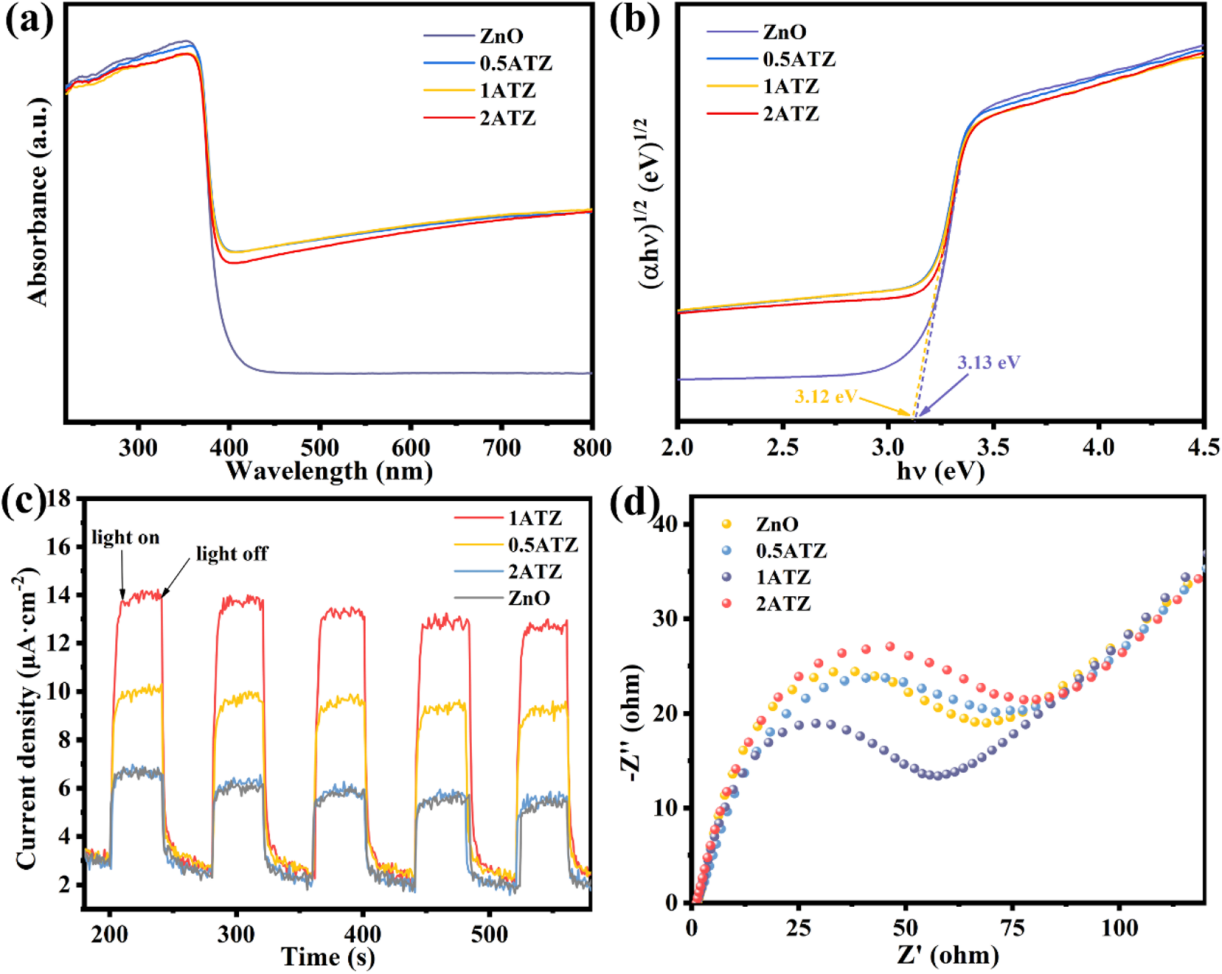
(a) UV-vis absorption spectra and (b) Tauc plots to estimate the band gap energies of ZnO and *x*ATZ composites. (c) Transient photocurrent responses (d) EIS plots of. ZnO and *x*ATZ.

UV-vis diffuse reflectance spectroscopy (UV-vis DRS) was utilized to investigate the optical properties of the photocatalysts. ZnO (Fig. 8a) has a distinct UV absorption peak at 360–390 nm, whereas MXns (Fig. S9†) shows full-spectrum absorption properties due to its pure black coloration with no distinct absorption fringes in the whole region (220–800 nm).^46^ Due to the low content of reduced Ag, no obvious surface plasmon resonance peaks about the singlet Ag appeared in the solid-state UV spectra.^48^ After the introduction of MXns into ZnO, the optical absorption intensity of the composite samples in the (400–800 nm) visible region was significantly enhanced due to the full-spectrum absorption of MXns. This means that the *x*ATZ composites can fully capture photons in the entire spectral range, and although the improved light trapping ability in the visible range cannot enhance the electron transport, the light energy can be converted into heat energy due to the superior photothermal conversion properties of MXns, which can promote the catalytic reaction kinetics and enhance the photocatalytic CO_2_RR performance. Tauc plots were made according to equation (*Ahν*)^*n*^ = *K*(*hν* − *E*_g_), as shown in Fig. 8b. The band gaps (*E*_g_) of ZnO and 1ATZ are 3.13 and 3.12 eV, which proves that the introduction of AT has almost no effect on the band gap of ZnO.^49^

Transient photocurrent response and electrochemical impedance spectroscopy (EIS) tests were performed using an electrochemical workstation to investigate the charge transfer efficiency of the photocatalysts. Fig. 8c shows that the photocatalysts all have a significant photocurrent response under light. The photocurrent intensity changed significantly under switching xenon lamps, and the current density of the photoelectrode was almost zero in the absence of light, and the current increased instantaneously when light was applied, indicating that the steeply rising current was generated by the introduction of light. 1ATZ had the highest photocurrent intensity, suggesting that the MXns was an effective co-catalyst to hinder the complexation of the photogenerated carriers and to enhance the photogenerated electron and hole separation in 1ATZ, corresponding to its most superior photocatalytic performance.^50^ The electrochemical performance and charge transfer of the photocatalysts were further illustrated using electrochemical impedance plots. As shown in Fig. 8d, 1ATZ has the smallest Nyquist circle radius, indicating the lowest charge transfer resistance and the highest transfer rate and separation efficiency of photogenerated carriers over the photocatalyst, which improves the photocatalytic CO_2_ reduction performance.^51^

**Fig. 9:**
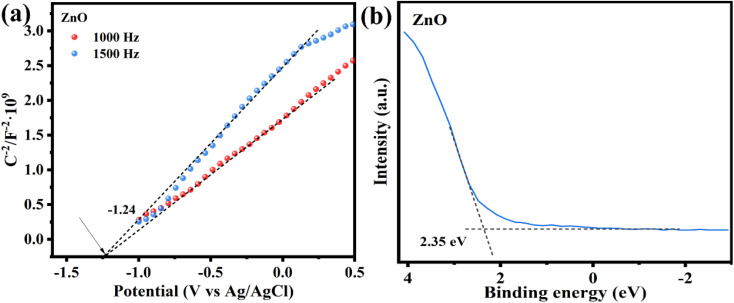
(a) Mott–Schottky plots at 1000 and 1500 Hz, (b) valence band spectra of ZnO.

To investigate the energy band structure of the photocatalyst, the Mott–Schottky (MS) curve test was performed. As shown in Fig. 9a, the slope of the curve is positive at 1000 and 1500 Hz, indicating that ZnO belongs to the n-type semiconductor material. The tangent intercept of the MS curve on the *x*-axis can be approximately equated to the flat-band band potential of the semiconductor material, which is −1.24 V (*vs.* Ag/AgCl, pH = 7) for ZnO.^52,53^ In n-type semiconductors, the conduction band potential is generally close to the flat band potential.^34^ The CB position of ZnO was calculated from the conversion equation of eqn (3), NHE potential to Ag/AgCl electrode potential.

3 *E*(NHE) = *E*(Ag/AgCl) − *E*_θ_ + 0.059 pH

When *E*_*θ*_ (Ag/AgCl, pH = 7) is 0.197 V, the *E*_CB_ of ZnO is −1.024 V (*vs.* NHE, pH = 7). Based on the results of UV-vis DRS (Fig. 8a), *E*_VB_ for ZnO was calculated to be 2.106 V (*vs.* NHE, pH = 7) using the *E*_VB_ = *E*_CB_ + *E*_g_ formula.^34^ The energy of the valence band position of ZnO relative to the Fermi energy level was measured as 2.35 eV by XPS valence band spectroscopy (Fig. 9b), and the valence band potential was calculated according to the conversion eqn (4):^54^

4 *E*(VB, NHE) = *φ* + *E*(VB, XPS) − 4.44

where *φ* is the power function of the XPS test instrument, *φ* = 4.2 eV. The valence band potential of ZnO (*E*_VB_) is 2.11 V. Combined with the *E*_g_ value of ZnO, the conduction band potential of ZnO (*E*_CB_) is calculated to be −1.02 V (*vs.* NHE, pH = 7), which is close to that estimated using the MS curve.

### 3.3 Photocatalytic mechanisms

**Fig. 10:**
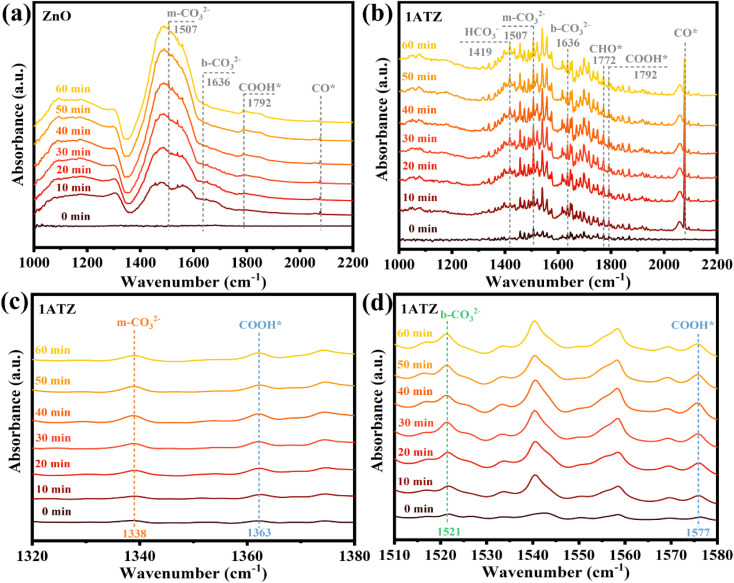
*In situ* DRIFTS spectra of (a) ZnO (b–d) 1ATZ in the presence of CO_2_/H_2_O under light irradiation for 0, 10, 20, 30, 40, 50, and 60 min.

*In situ* diffuse reflectance infrared Fourier transform spectroscopy (DRIFTS) tests were performed to study the adsorption and activation of CO_2_ molecules on the surface of photocatalysts and to understand the pathway of photocatalytic CO_2_ reduction. The *in situ* spectroscopy of ZnO and 1ATZ were recorded in darkness and after 10, 20, 30, 40, 50, and 60 min of turning on the light under the CO2 atmosphere. As shown in Fig. 10b, symmetric stretching and asymmetric stretching vibrations belonging to HCO_3_^−^, which is an existent form of adsorbed CO_2_, were observed at 1419 and 1456 cm^−1^, and the peaks of HCO_3_^−^ were not evident in ZnO (Fig. 10a). The appearance of the peaks of the monodentate carbonate (m-CO_3_^2−^) at 1338, 1507, and 1558 cm^−1^, once again demonstrated that CO_2_ underwent chemical adsorption (Fig. 10(a–c)).^55^ Meanwhile, the peaks at 1636 and 1521 cm^−1^ are attributed to bidentate carbonates (b-CO_3_^2−^).^56^ The intensity of the FTIR peaks in the 1ATZ spectra regarding the adsorption of CO_2_ varied more significantly with time than that of ZnO, indicating that the introduction of AT can enhance the ability of the photocatalysts to adsorb CO_2_. On the contrary, the intensities of all FTIR peaks on ZnO were very low, which proved that ZnO itself had a weak adsorption of CO_2_. The four peaks at 3728, 3704, 3625, and 3597 cm^−1^ bands correspond to the stretching vibration of –OH (Fig. S10†), and the generation of surface hydroxyl groups is attributed to the dissociative adsorption of H_2_O.^57^ The strong interaction between the catalyst and water molecule leads to the competition between H_2_O and CO_2_ for the active sites on the catalyst surface, which reduces the performance of CO_2_ photoreduction.^41^ As shown in Fig. S8,† after maintaining in a CO_2_ atmosphere for 5 min in the dark state, the surface hydroxyl groups of 1ATZ were observed to be less than that of pure ZnO, indicating that the MXns co-catalyst can effectively inhibit the competitive adsorption of H_2_O, which in turn facilitates the adsorption of CO_2_ and the CO_2_RR reaction.

After illumination, new spectral peaks appeared in the infrared spectra of both the original and composite samples. The peaks appearing at 1792, 1577, and 1363 cm^−1^ are attributed to CO_2_RR intermediate COOH* (* represents the adsorption state on the surface of the photocatalyst) for the generation of various types of carbon-based fuels.^58,59^ The appearance of the characteristic peaks of CHO* (1772 cm^−1^) and CO* (2077 cm^−1^) indicates that the reduction products contain CH_4_ and CO, with CHO* and CO* are the key intermediates for the generation of CH_4_ and CO, respectively.^60^ It is noteworthy that the COOH* peak is more prominent in the local amplified infrared spectrum of 1ATZ (Fig. 10d), and the intensity of the characteristic peaks gradually enhanced with the extension of the light time, indicating that more CO_2_ molecules were adsorbed and activated on the surface of 1ATZ under the light, and the intermediates COOH* continues to be produced and accumulated. The spectral peaks corresponding to CHO* and CO* increased with the reaction time and the intensity of the CO_2_ adsorption bands at 1772, 1577, and 1344 cm^−1^ increased gradually, indicating that the continuous adsorption and activation of CO_2_ on the 1ATZ samples favorable for the photocatalytic CO_2_RR. In conclusion, the introduction of AT significantly enhanced the adsorption and activation of CO_2_.

Based on the mentioned results and analyses, the pathway of 1ATZ photocatalytic CO_2_ reduction may be eqn (5)–(12). CO_2_ molecules are first adsorbed on the surface of the photocatalyst, and then 
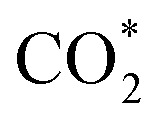
 interacts with H^+^ divorced from adsorbed water and photogenerated electrons to form an intermediate product, COOH*. COOH* gains a pair of electrons and H^+^ which are further dehydrated to produce CO*. Finally, CO* desorbs from the photocatalyst surface to produce CO molecules. On the other hand, a small amount of CO* generates CHO* and CH_3_O* by hydrogenation reaction, and finally forms CH_4_.5
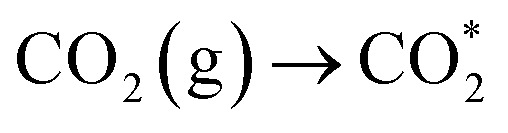
6
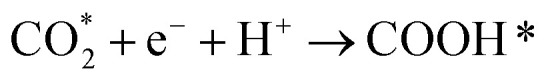

7 COOH* + e^−^ + H^+^ → CO* + H_2_O

8 CO* → CO (g)

9 or CO* + e^−^ + H^+^ → CHO*

10 CHO* + e^−^ + H^+^ → CH_2_O*

11 CH_2_O* + e^−^ + H^+^ → CH_3_O*

12 CH_3_O* + e^−^ + H^+^ → CH_4_ (g) + O*

Based on the energy band structure and photocatalytic reaction pathways, a possible photocatalytic mechanism for the ternary heterostructure of ATZ is proposed. According to the literature, the *E*_f_ values of Ti_3_C_2_T_*x*_ with terminal groups –F and –O are 0.18 V and 0.71 V (*vs.* NHE, pH = 7),^34^ which are both much lower than the conduction band position of ZnO, and thus the photoexcited electrons tend to be transferred from ZnO to MXns across the heterojunction interface. The Fermi energy level of pure MXns is lower than the *E*(CO_2_/CO) and *E*(CO_2_/CH_4_) conversion energies, so CO_2_ cannot be reduced on its surface.^61^ It is shown that the *E*_f_ of n-type semiconductors is close to the bottom of the conduction band, and after the Mott–Schottky curve and valence band spectrum tests, the Fermi energy level of ZnO is much higher than that of MXns before contact.

**Fig. 11:**
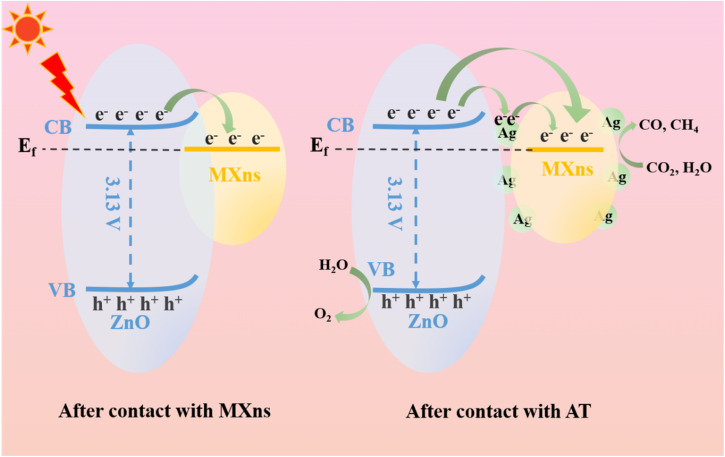
Schematic illustration of charge transfer route and photocatalytic mechanism of ATZ heterojunction.

Consequently, the difference in Fermi energy levels between ZnO and MXns drives the electrons to migrate from ZnO to MXns to balance the *E*_f_ of the two materials after contact. As the interface between ZnO and MXns is in direct contact, the *E*_f_ of ZnO is positively displaced and the *E*_f_ of MXns is negatively displaced, while the *E*_f_ finally reaches equilibrium. During the equilibrium process, the energy bands of ZnO will bend upward, creating a Schottky barrier, as shown in Fig. 11. Therefore, two possible electron migration modes are proposed.^62^ When there is no silver cluster at the interface between ZnO and MXns, the photogenerated electrons on ZnO will rapidly migrate to the surface of MXns due to the good electron trapping ability of MXns and the close contact of the interface between ZnO and MXns, which greatly promotes the separation of the photogenerated carriers. At the same time, the Schottky barrier prevents the return flow of photogenerated electrons and inhibits the complexation of electron–hole pairs. The accumulated charges on the MXns surface can reduce the adsorbed CO_2_ molecules to CO and CH_4_ gases at this potential equilibrium. When Ag is present at the interface between ZnO and MXns, electrons are first transferred from ZnO to Ag and then from Ag to the MXns surface. Finally, the electrons are gathered on the AT surface for photocatalytic CO_2_ reduction.

## 4. Conclusion

In this paper, AT composites were prepared by self-reduction on the Ti_3_C_2_T_*x*_ surface. The nanoflower ZnO was successfully coupled with the composite AT by an electrostatic self-assembly process to form ATZ ternary heterojunction photocatalysts, which were used for photocatalytic CO_2_ reduction. The prepared ATZ ternary heterojunction composites have excellent photocatalytic performance and photocatalytic CO_2_ reduction efficiency. The ATZ photocatalysts showed the highest CO and CH_4_ reduction efficiencies of 11.985 μmol g^−1^ h^−1^ and 0.768 μmol g^−1^ h^−1^, respectively, and the CO_2_ conversion was 3.35 times higher than that of pure ZnO, which also showed excellent performance among similar catalysts. The prepared composite photocatalysts showed high stability after four cycling experiments. The presence of MXns and Ag provides ZnO nanomaterials with more adsorption active sites and reaction centers for CO_2_ adsorption and activation. The unique Schottky heterojunction structure of ATZ promotes the separation and migration of photogenerated carriers, which significantly enhances the photocatalytic reduction efficiency. This study provides a new strategy for the design and preparation of efficient and stable semiconductor photocatalysts, which also leads up new directions for the preparation of MXene-based photocatalytic composites.

## Conflicts of interest

The authors declare that they have no known competing financial interests or personal relationships that could have appeared to influence the work reported in this paper.

## Supplementary Material

RA-014-D4RA01985G-s001
