# Supplementary material for: Accelerating carrier separation to boost the photocatalytic CO2 reduction performance of ternary heterojunction Ag–Ti3C2Tx/ZnO catalysts
Source: RSC Adv. 2024 Apr 25;14(20):13719–33. doi: 10.1039/d4ra01985g (PMC11044907; doi:10.1039/d4ra01985g)
Supplement: RA-014-D4RA01985G-s001 [file RA-014-D4RA01985G-s001.pdf]

Electronic Supplementary Material (ESI) for RSC Advances.
This journal is © The Royal Society of Chemistry 2024

Supporting information

Supplemental figures

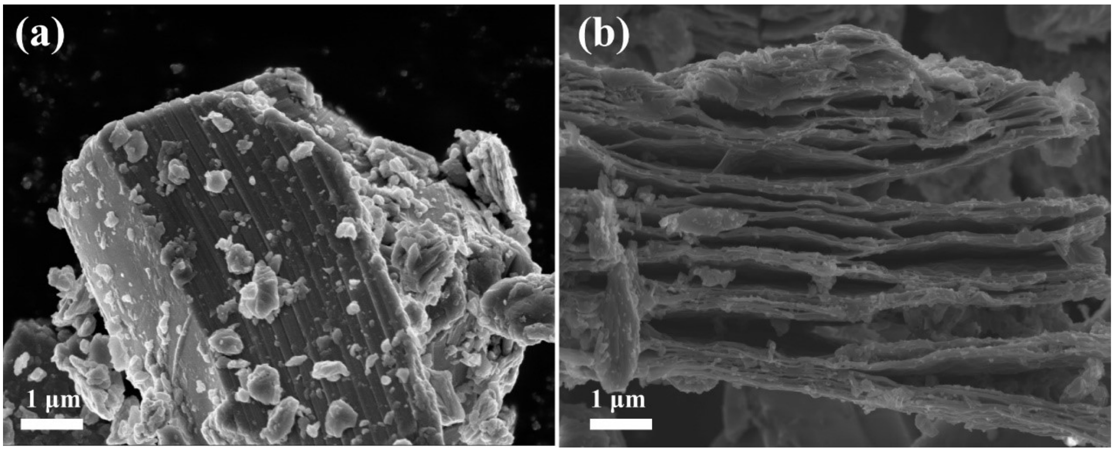

Fig. S1 SEM images of (a) Ti_3AlC_2 and (b) MX.

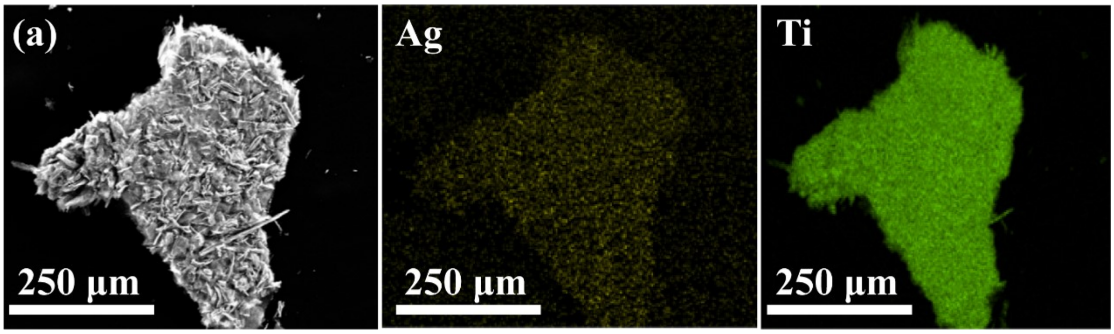

Fig. S2. SEM-EDS-Mapping images of Ag and Ti elements over AT sample.

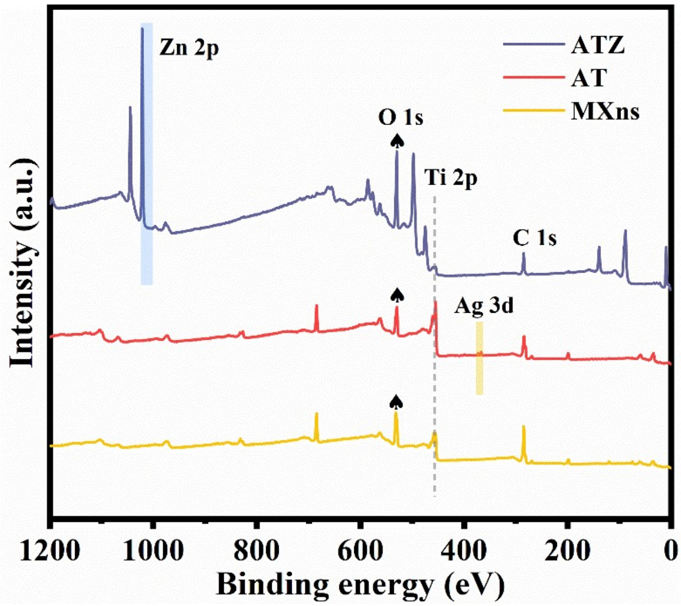

Fig. S3. XPS full spectra of the MXns, ZnO, and ATZ

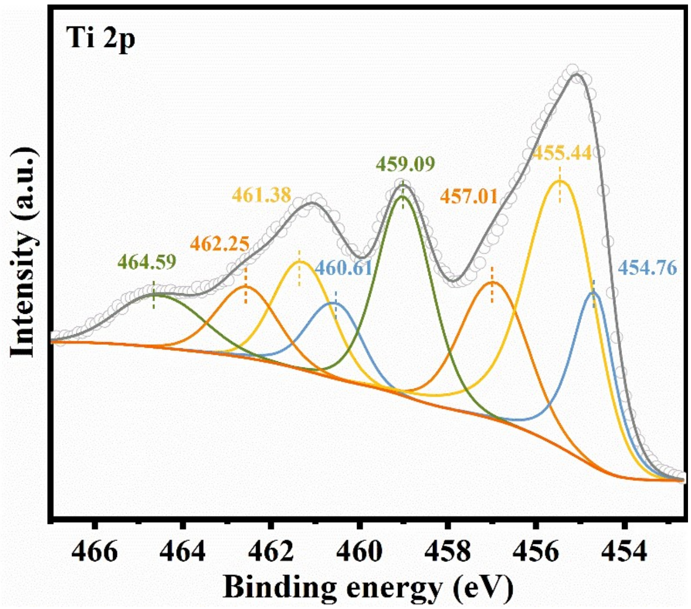

Fig. S4. XPS spectra of Ti 2p for AT.

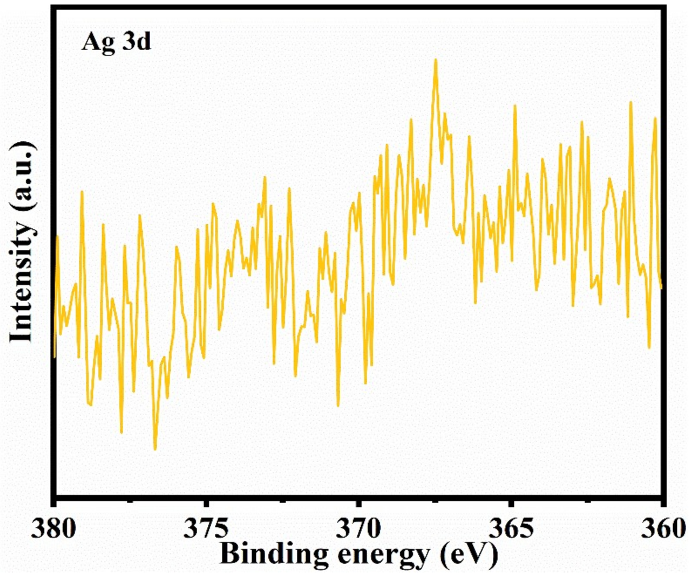

Fig. S5. XPS spectra of Ag 3d for 1ATZ.

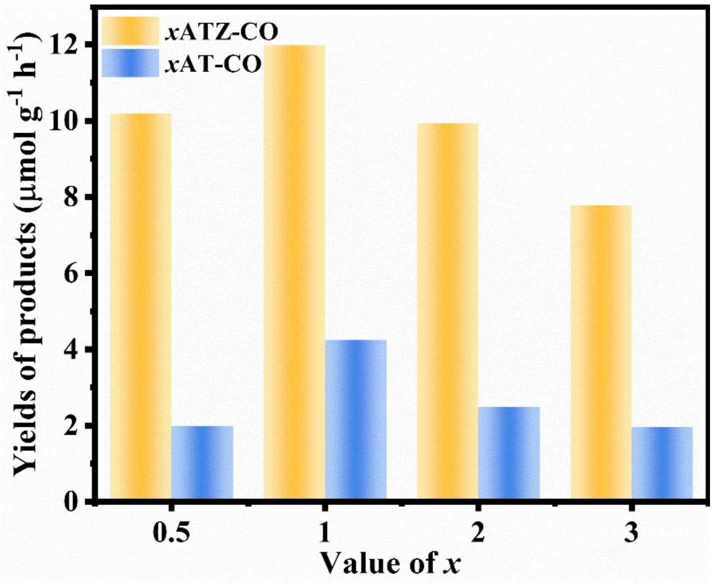

Fig. S6. CO yield plots of xAT and xATZ.

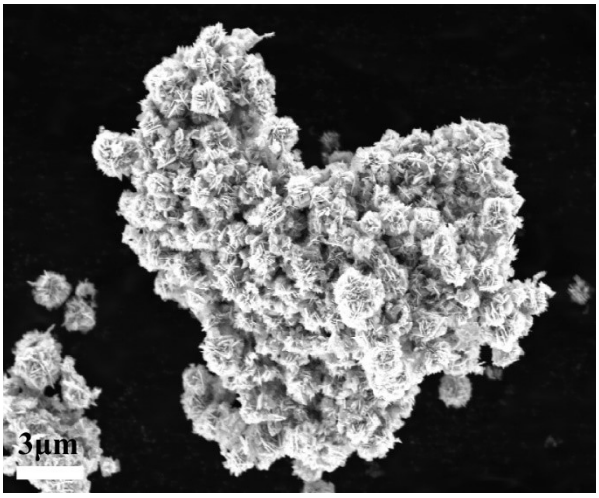

Fig. S7. SEM images of 2ATZ.

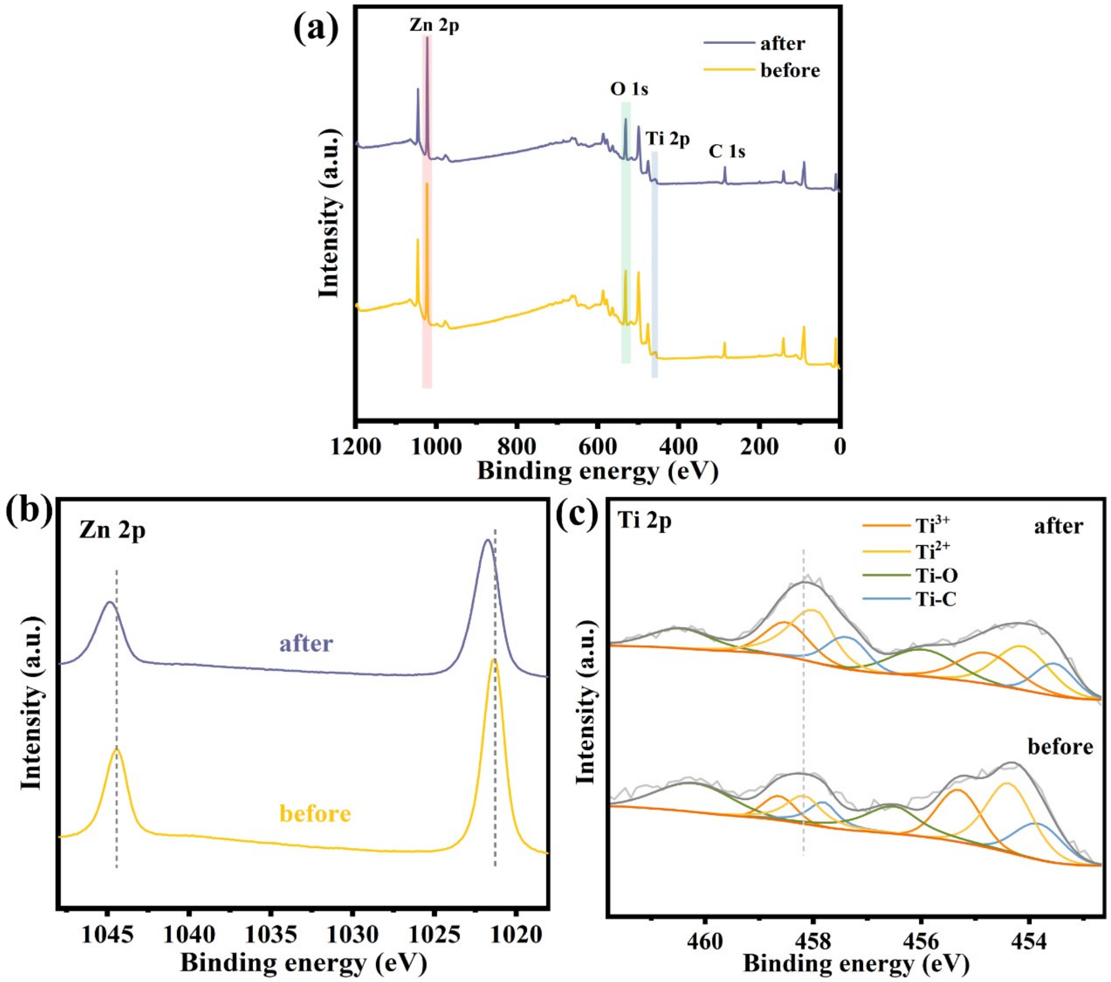

Fig. S8. XPS spectra of (a) full spectra (b) Zn 2p (c) Ti 2p for 1ATZ before and after use.

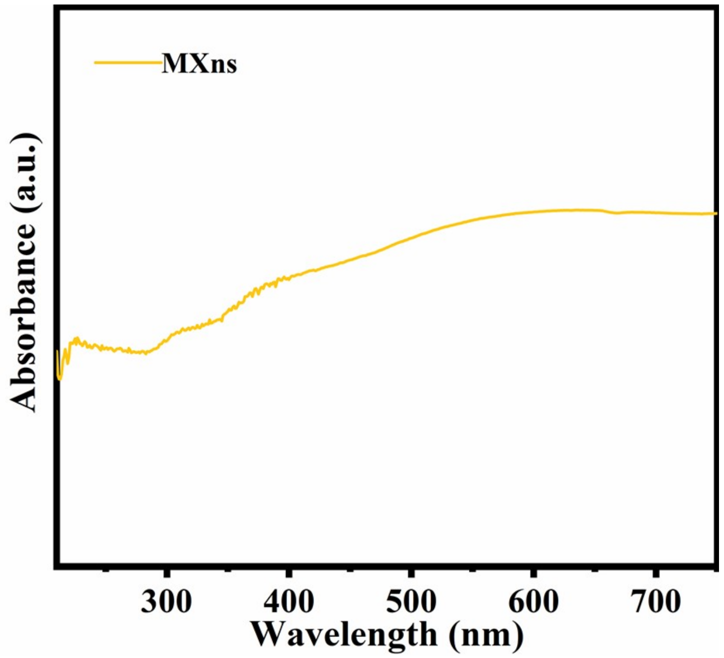

Fig. S9. UV–vis absorption spectra of MXns.

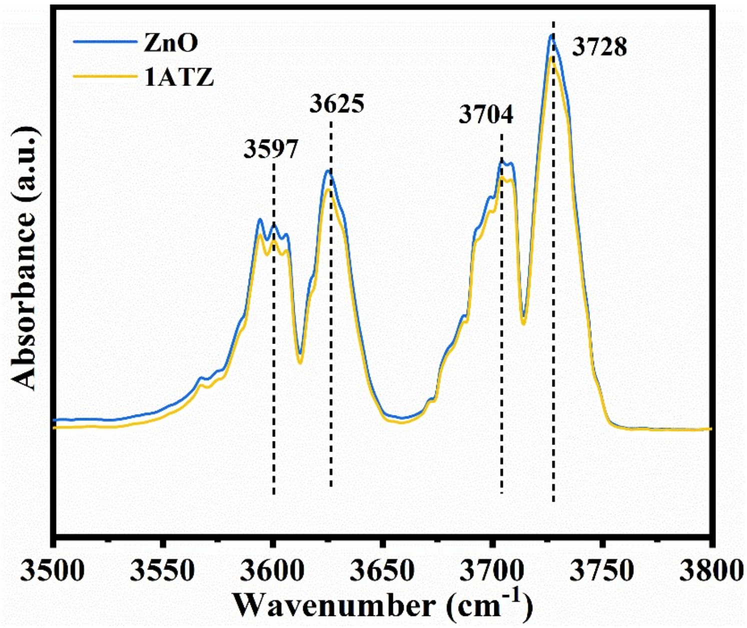

Fig. S10. In-situ DRIFTS spectra of CO_2 adsorption on 1ATZ and ZnO in the presence of CO_2/H_2O for 5 min in the dark.

Table S1. Specific surface area and pore size of the samples

Samples	Specific surface area (SSA) ($m^2 \cdot g^{-1}$)	Pore size (nm)	Pore volume ($cm^3 \cdot g^{-1}$)
ZnO	91.794	7.304	0.212
MXns	81.557	4.573	0.083
1ATZ	153.298	7.275	0.299

[1] C. Yang, Q. Tan, Q. Li, *et al.*, 2D/2D Ti_3C_2 MXene/g-C_3N_4 nanosheets heterojunction for high efficient CO_2 reduction photocatalyst: Dual effects of urea, Appl. Catal. B-Environ., 268 (2020) 118738.

[2] Q. Tang, Z. Sun, S. Deng, *et al.*, Decorating g-C_3N_4 with alkalinized Ti_3C_2 MXene for promoted photocatalytic CO_2 reduction performance, Journal of Colloid and Interface Science, 564 (2020) 406-417.

[3] W.Z. Liu, M.X. Sun, Z.P. Ding, *et al.*, Ti_3C_2 MXene embellished g-C_3N_4 nanosheets for improving photocatalytic redox capacity, Journal of Alloys and Compounds, 877 (2021).

[4] B. Yu, Y. Zhou, P. Li, *et al.*, Photocatalytic reduction of CO_2 over Ag/TiO_2 nanocomposites prepared with a simple and rapid silver mirror method, Nanoscale, 8 (2016) 11870-11874.

[5] F. He, B. Zhu, B. Cheng, *et al.*, 2D/2D/0D TiO_2/C_3N_4/Ti_3C_2 MXene composite S-scheme photocatalyst with enhanced CO_2 reduction activity, Appl. Catal. B-Environ., 272 (2020) 119006.

[6] W.N. Shi, R. Zhang, J.C. Wang, *et al.*, One-pot hydrothermal preparation of Ni and I co-doped brookite-anatase TiO_2 nanoparticles with remarkably enhanced photoreduction activity of CO_2 to CH_4, Journal of Catalysis, 429 (2024).

[7] T.T. Li, R. Tao, Y.X. Wang, *et al.*, Construction of bismuth oxide iodide (BiOI)/zinc titanium oxide (Zn_2TiO_4) p-n heterojunction nanofibers with abundant oxygen vacancies for improving photocatalytic carbon dioxide reduction, Journal of Colloid and Interface Science, 655 (2024) 841-851.

[8] R.A. Mahmud, K.A. Ali, L.K. Putri, *et al.*, ZnO with engineered surface defects as a competent photocatalyst for CO_2 photoreduction into valuable fuels under simulated solar light irradiation, Journal of Environmental Chemical Engineering, 11 (2023).

Materials	Light source	CO production rate (μmol/g/h)	CH_4 production rate (μmol/g/h)	references
g-C_3N_4/Ti_3C_2	300 W Xe lamp	5.19	0.04	[1]
g-C_3N_4/Ti_3C_2	300 W Xe lamp	2.24	0.05	[2]
g-C_3N_4/Ti_3C_2	300 W Xe lamp	2.67	0.66	[3]
1.5% Ag/TiO_2	300 W Xe lamp	/	1.40	[4]
TiO_2/C_3N_4/Ti_3C_2	300 W Xe lamp	4.39	1.20	[5]
2Ni/9I-TiO_2	300 W Xe lamp	0.233	10.55	[6]
BiOI/Zn_2TiO_4	300 W Xe lamp	9.10	/	[7]
ZnO nanoparticles	500 W Xe lamp	/	<0.043	[8]
Ag-$Ti_3C_2T_x$/ZnO	300 W Xe lamp	11.985	0.768	This work

Table S2. Comparison of photocatalytic reduction activities of similar catalysts
